# Beyond Correlation in the Detection of Climate Change Impacts: Testing a Mechanistic Hypothesis for Climatic Influence on Sockeye Salmon (*Oncorhynchus nerka*) Productivity

**DOI:** 10.1371/journal.pone.0154356

**Published:** 2016-04-28

**Authors:** Michael D. Tillotson, Thomas P. Quinn

**Affiliations:** University of Washington School of Aquatic and Fishery Sciences, Seattle, Washington, United States of America; Technical University of Denmark, DENMARK

## Abstract

Detecting the biological impacts of climate change is a current focus of ecological research and has important applications in conservation and resource management. Owing to a lack of suitable control systems, measuring correlations between time series of biological attributes and hypothesized environmental covariates is a common method for detecting such impacts. These correlative approaches are particularly common in studies of exploited fish species because rich biological time-series data are often available. However, the utility of species-environment relationships for identifying or predicting biological responses to climate change has been questioned because strong correlations often deteriorate as new data are collected. Specifically stating and critically evaluating the mechanistic relationship(s) linking an environmental driver to a biological response may help to address this problem. Using nearly 60 years of data on sockeye salmon from the Kvichak River, Alaska we tested a mechanistic hypothesis linking water temperatures experienced during freshwater rearing to population productivity by modeling a series of intermediate, deterministic relationships and evaluating temporal trends in biological and environmental time-series. We found that warming waters during freshwater rearing have profoundly altered patterns of growth and life history in this population complex yet there has been no significant correlation between water temperature and metrics of productivity commonly used in fisheries management. These findings demonstrate that pairing correlative approaches with careful consideration of the mechanistic links between populations and their environments can help to both avoid spurious correlations and identify biologically important, but not statistically significant relationships, and ultimately producing more robust conclusions about the biological impacts of climate change.

## Introduction

The response of animal populations, biological communities, and entire ecosystems to global climate change has become a dominant theme in the field of ecology [1]. Observed warming in recent decades has provided opportunities for empirical, *in situ* research on the biological impacts of climate change across a broad geographic and taxonomical range [2,3]. Biological responses to these physical changes have been diverse, documented in a wide variety of species including mammals, birds, fishes, amphibians, plants, involving altered spatial distribution, timing of key life history events, demographics, growth and survival [3–6]. Despite abundant evidence that climate change affects species’ distribution, growth and phenology, the implications of these effects for population productivity and persistence are less clear. Even in the absence of a clearly specified mechanism, correlations between environmental variables and population productivity are frequently reported, and may serve as the basis for predicting future abundances [7]. However, a variety of intrinsic ecological processes may mask or confound such relationships [8,9]. Population level responses including phenotypic and demographic plasticity, evolution, and altered behavior may affect observed species-environment relationships [3,10]. In populations subject to significant density dependence, compensatory growth or survival may moderate or exacerbate the impacts of environmental change [11,12]. Furthermore, environmental change is often spatially or temporally heterogeneous and therefore a negative impact at one life-history stage may prove beneficial in later stages (e.g. [13]). It is therefore not surprising that environment-productivity correlations frequently break down when confronted with new data [7].

Understanding the ongoing and future impacts of climate change is especially pressing for commercially important fish species because environmentally driven changes in productivity can have severe economic, social and cultural consequences [14]. For example, Pacific salmon (*Oncorhynchus* spp.) populations have fluctuated in response to large-scale climate variability in recent decades with impacts on fishermen felt around the Pacific Rim [15–17]. Across the North Pacific salmon populations tend to display prolonged periods of high and low productivity, often correlated with climate indices [18,19]. However, the mechanisms by which large scale climate forcing influences salmon populations are not well understood, and individual populations often diverge significantly from the average pattern [10]. In part this among-population variability can be explained by strong population-specific density dependence and differences in freshwater habitats. Additionally, life history variability may result in diverse responses to common environmental conditions [20]. In these fishes the timing of key ontogenetic shifts including seaward migration (smolting) and homeward migration (maturation) depends on size and growth rate [21]. As a result, even salmon from the same population and cohort may be subject to disparate environmental conditions and schedules of mortality. For example, individuals spawned in the same year but migrating to sea after one or two years of freshwater residence will enter the ocean in different years, and thus experience different environmental conditions at sea. They will also differ in size during the stressful ocean entry phase, and may spend different lengths of time at sea prior to maturation and return [22]. Climate, life history and productivity are therefore interdependent, and accounting for these relationships is necessary to interpret past variability and predict future responses to environmental change in species such as salmon that display complex life histories [23].

**Fig 1:**
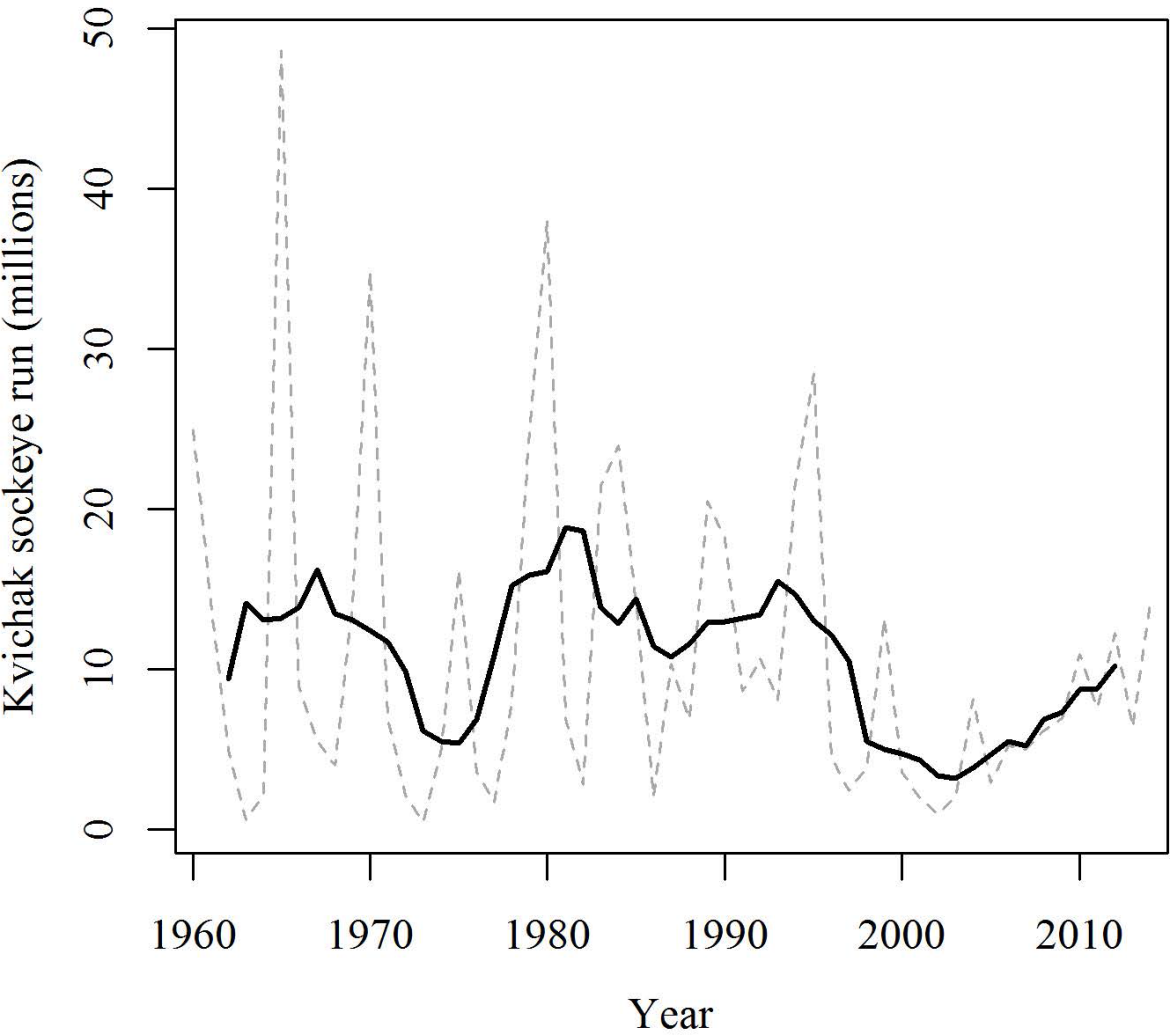
Kvichak River annual sockeye run size and 5-year moving average, 1960–2014.

The life history patterns of Pacific salmon exemplify the complex links between climate and population productivity, and the rich data collected on the commercially valuable species allow us to test critical hypotheses regarding these links. In this study we examine over five decades of data on the largest complex of sockeye salmon populations in the world, produced in the Kvichak River basin in Bristol Bay, Alaska. Beginning in the late 1990s sockeye salmon from the Kvichak River system experienced a decrease in productivity at the same time that many other parts of the Bristol Bay population complex increased (Fig 1) [24]. Although asynchronous changes in productivity among the major rivers is characteristic of the Bristol Bay stock-complex[25], the decline was nonetheless alarming. With significant warming observed in the region during the past two decades [26] there has been much speculation about what role climate change could be playing in this apparent decline, and what characteristics of the Kvichak River population might make it particularly vulnerable to warming [10,27].

Lew [28] proposed a mechanistic hypothesis for climate induced productivity declines in the Kvichak River system; increased juvenile growth opportunity associated with climate warming has resulted in a shift from predominantly age-2 to age-1 seaward migrants. In turn, average marine survival has declined because these younger migrants are smaller than they would have been had they remained in the lake for an additional year, and marine survival in salmon is generally size-dependent. Juvenile growth in the system is also density dependent [29] so a positive feedback occurs with even faster growth of juveniles as abundance declines, enhancing their tendency to leave the lake young, hence small. The Kvichak River could be particularly susceptible to this change because–more than other Bristol Bay rivers–large returns have historically been dominated by age-2 smolts [27]. Overall, this hypothesis predicts a negative correlation between temperature experienced during freshwater rearing and population productivity; an environment-productivity relationship that can be easily evaluated using a typical correlative approach.

Alternatively, several nested hypotheses can be tested to shed light on the mechanisms relating climate change and productivity. These intermediate hypotheses include: 1) a positive relationship between fry growth and growing season temperature, 2) a negative relationship between fry growth and average age at seaward migration, 3) a positive relationship between marine survival and smolt age, and 4) marine survival accounts for an appreciable proportion of variation in total lifetime survival. In this study we utilized a multi-step approach to test the hypothesis that warming waters and the earlier age at juvenile migration are indeed resulting in reduced productivity of Kvichak River sockeye. First, we described temporal trends in a suite of environmental variables and population attributes to evaluate their general consistency our hypotheses and to identify potentially confounding factors. We next modeled the hypothesized intermediate, deterministic relationships to test the strength of the mechanistic link between climate and productivity. Finally, in order to allow for comparison between the correlative and mechanistic approaches we evaluated the influence of climate change on sockeye salmon productivity by incorporating growing season temperature as an environmental covariate in a stock- recruit model.

## Materials and Methods

### Study Site and Population Characteristics

**Fig 2:**
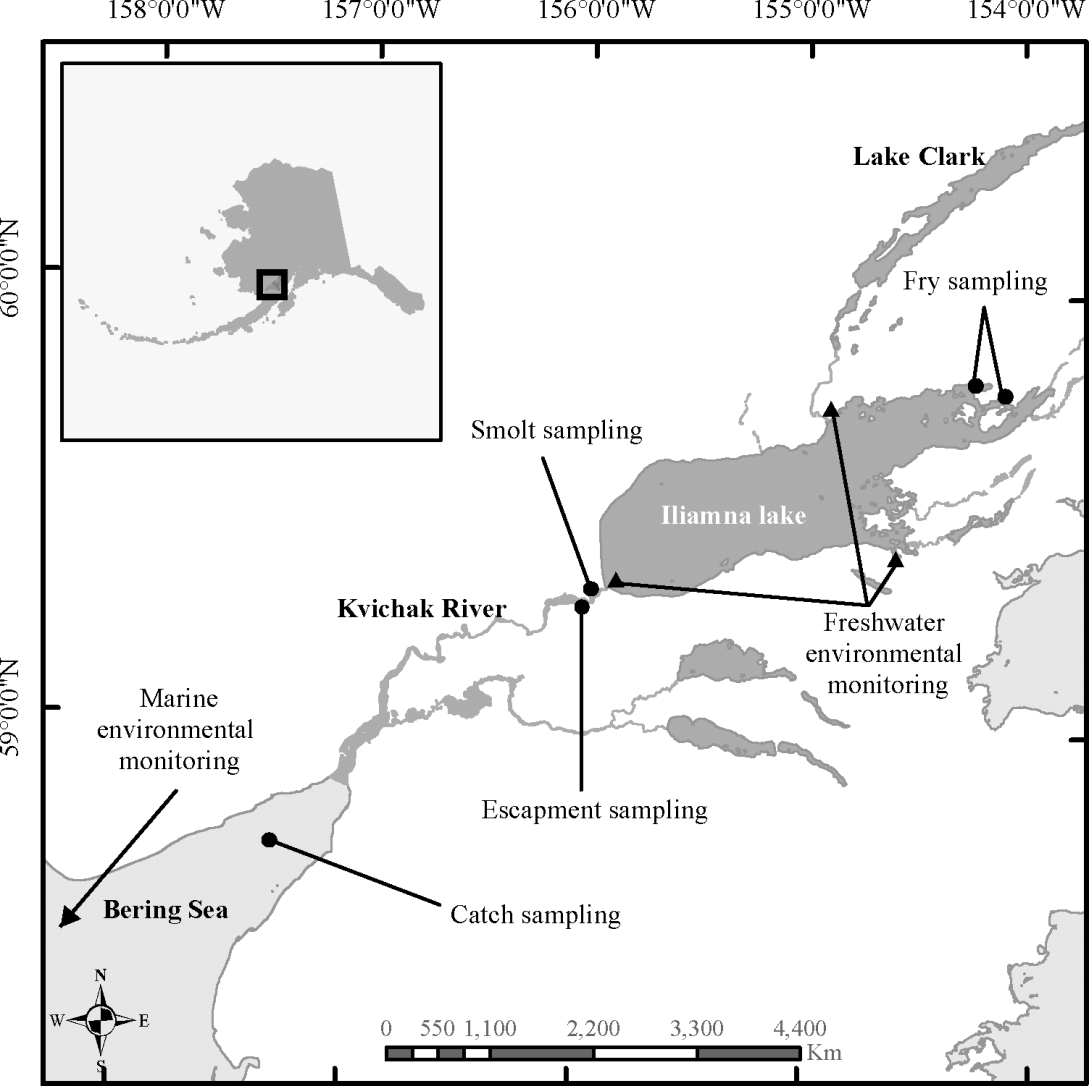
Kvichak River drainage, Alaska showing biological and environmental data collection sites. Spatial data retrieved from the USGS National Hydrography Dataset (nhd.usgs.gov).

The river systems that drain to Bristol Bay in southwest Alaska comprise the world’s largest sockeye salmon producing region (Fig 2). Intensive commercial fishing with gillnets has occurred for over 100 years in five districts located near river mouths within the bay [22]. Each river drains one or more large lakes where sockeye salmon generally rear for one or two years before migrating to the Bering Sea and Gulf of Alaska [30]. Iliamna Lake is the largest in the watershed (and in Alaska) with a surface area of approximately 2622 km^2^. The Kvichak River drains from Iliamna Lake approximately 30 km to Kvichak Bay and the Naknek-Kvichak fishing district where most of the population’s fishery mortality occurs [29].

**Fig 3:**
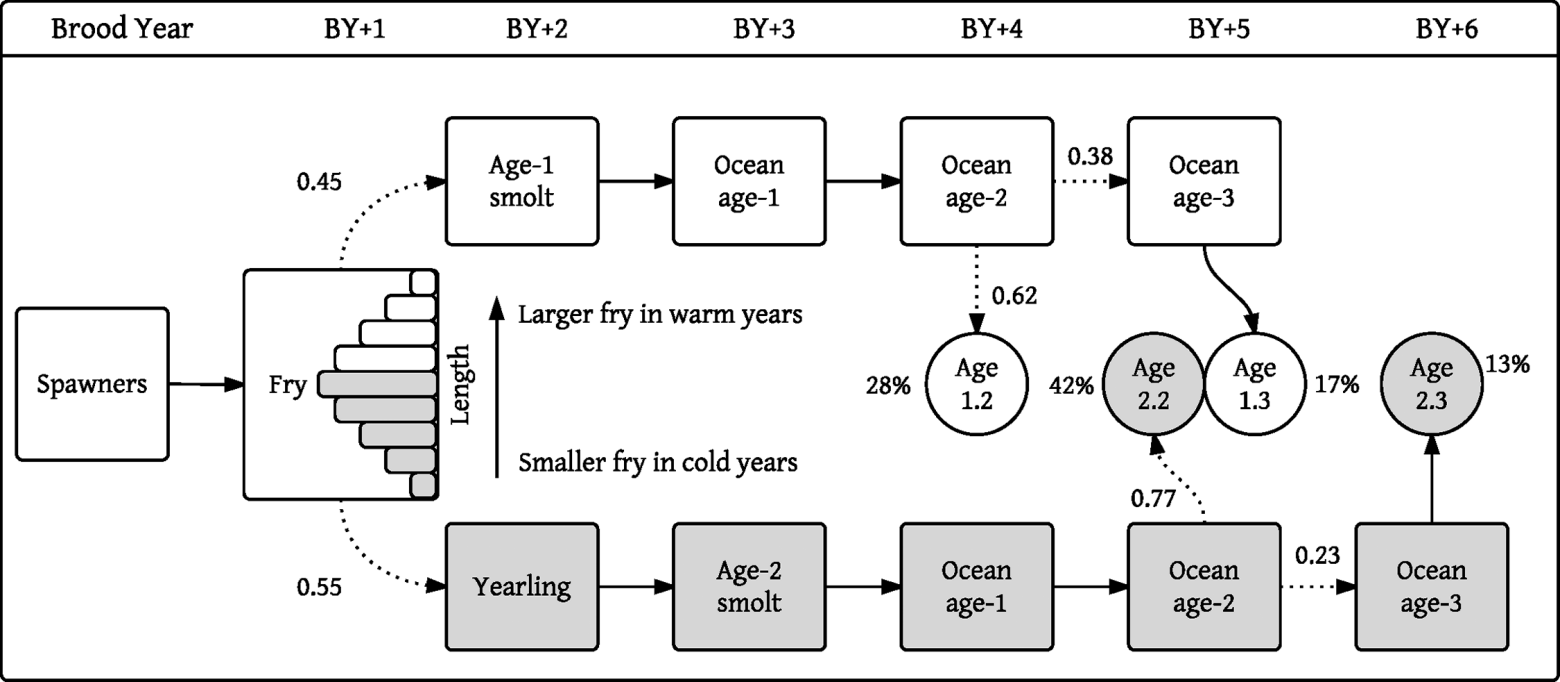
Kvichak sockeye salmon life-history and main life-history pathways. Percentages give long-term average age composition. Dotted lines indicate variable life history pathways and are labeled with the long-term average proportions of individuals following each pathway. Fry length frequency histogram shows relationship between freshwater growth and smolt age.

Sockeye salmon return to the Kvichak River during June and July and spawn on the beaches of Iliamna Lake or in its many tributaries throughout the summer and early autumn. Fry emerge the following spring and enter the lake where they feed–almost always for one or two years–before migrating to sea [31]. Ocean residence typically lasts two or three years, though a small fraction of the males returns after only one year. Thus, the majority of Kvichak River sockeye mature at ages designated 1.2 (i.e. one year of freshwater rearing and two years at sea), 1.3, 2.2 and 2.3 (note that the winter of incubation is not accounted for in these age distinctions so a fish returning at age 1.2 is 4 calendar years old; see Fig 3). The age composition of the Kvichak River population has varied through time [32]. Both seaward and homeward migration are at least somewhat dependent on growth, and patterns in age composition are therefore thought to reflect both external (i.e. environmental) and internal (i.e. density dependent) processes [21,22,29].

### Data collection and processing

For salmon populations, productivity is generally measured in terms of returns per spawner (R/S); the number of salmon surviving natural mortality and returning to spawn or be caught as adults that was produced by the parental generation in a given previous year (i.e. brood year). Because age at maturity varies sockeye salmon of the same cohort will return to spawn over several years. To accurately measure productivity it is thus necessary to know both the abundance and age of returning fish. Sampling of catch and escapement has been conducted in Bristol Bay since the mid-1950s and is reported annually by the Alaska Department of Fish and Game (ADF&G) in regional management reports (e.g. [33,34]). Catch is enumerated daily by dividing the total weight of landings by average fish weight for each district. Fish that survive through the fishing district are termed ‘escapement’ and are enumerated by counting towers located on the major rivers in Bristol Bay [35]. Age can be determined from scales that are collected from sample of the catch at processing facilities and from the salmon that escaped the fisheries using beach seines near the counting towers. Age composition samples are expanded based on total catch and escapement to estimate the total number of returning fish of each age [33]. Catch data as reported by ADF&G did not always account for potential interceptions of Kvichak River fish in non-terminal fisheries including the South Alaska Peninsula, on the high seas, and other Bristol Bay districts. Run reconstructions using retrospective genetic studies have made it possible to correct bias in productivity estimates that can be introduced by these interceptions. We therefore use an updated version of the corrected catch and escapement data set described in Cunningham et al. [36] to calculate returns by age class.

Brood tables were constructed by assigning returns to the year in which they were spawned. Abundance and age composition of catch and escapement have been estimated using consistent methods since 1956 and validated data were available through the 2014 return. The large majority of fish (>99%) return by age six and we were therefore able to construct brood tables and estimate productivity for brood years 1956–2009. Brood tables were used to calculate several population attributes. First, total R/S was calculated for each brood year. Next, to account for potential declines in productivity at high spawner abundances (i.e. density dependence) a density-independent index of productivity was developed. We fitted a Ricker [37] model to brood table data using a linear regression of the natural logarithm of R/S on spawner abundance. We then extracted the model residuals as an index of density-independent productivity (R/S index [38]). In order to examine freshwater and marine productivity independently, in years where data on seaward migrating juveniles were available (see below) total smolts per spawner (S/S) and smolt-to-adult survival (SAS) were also calculated.

In addition to metrics of abundance and productivity we also sought to describe age composition which may be sensitive to environmental variability and could therefore be influenced by climate change. Because on average four age classes– 1.2, 1.3, 2.2 and 2.3 –make up over 99% of the return (minimum of 97.8% in our time-series) both freshwater and marine age can be considered as a binary responses, and thus each is described by a single proportion. For each brood year freshwater age composition of fish surviving to maturity is as the total return of freshwater age-1 individuals divided by the total return while marine age composition is described by the proportion of ocean age-2 fish in the return.

Efforts to annually monitor the juvenile life-history phase of Kvichak River sockeye began in 1956. Initially, smolts (seaward migrating juveniles) were sampled throughout the spring migration season at a location just downstream of Iliamna Lake on the Kvichak River. Over the history of the sampling program consistent methods have been used to estimate the age composition, mean age and mean weight of the migrating smolts on a daily basis; daily values are then aggregated in to seasonal averages. We calculated smolt condition factor as the residuals of a linear regression of the natural logarithms of weight and length across all smolt years with available data [39]. Daily relative abundance is used to characterize migration timing and can be used to estimate benchmarks including median migration date. Additionally, smolt abundance has been estimated in most years. In total, age-specific smolt abundance estimates are available for 1956–2001 and 2008–2014 [27,40]. While age and size sampling methods have remained consistent the smolt enumeration methodology has varied over time and included standardized fyke net sampling, hydroacoustics, and modeling. Several efforts have been made to correct for the changes in sampling methods [27,41]. We use the estimates given by Ruggerone and Link [27] and update them with data from recent annual reports (e.g. [40]). After accounting for the two or three year lag between brood year and migration year we estimated the total smolt production and juvenile age composition for 53 brood years: 1956–1998 and 2006–2012.

In addition to smolt sampling, the length and weight of sockeye salmon fry at the end of their first growing season in Iliamna Lake have been estimated annually since 1962. Fry are sampled using a standardized tow-netting procedure at two index areas in the eastern end of the lake (Pedro Bay and Knutson Bay; see Rich et al. 2009 for complete methodology). Spawning occurs primarily in this region of the lake and juveniles tend to migrate westward as they become older [31]. As a result the vast majority of individuals sampled were age-0; those spawned the previous autumn. Average fish lengths were weighted by the surface area of the sampling areas (a constant among years) and adjusted to a common date of 1 September using observed growth rates to account for variation in the precise sampling date among years [31]. The time series for fry length, after accounting for a one year lag between spawning and capture covers brood years 1961–2014, except for 1997 when sampling did not occur, for 52 years in total.

Although we are primarily interested in the influence of spring water temperature on population productivity, we recognized that environmental changes influencing other portions of the sockeye salmon life-history may also influence the population. To evaluate consistency in the magnitude and direction of environmental changes, we examined trends in a broad range of environmental variables that potentially influence Kvichak River sockeye salmon throughout their life-cycle. Water temperature mediates many important processes including growth, survival and phenology in salmon. Water temperatures collected at the outlet of Iliamna Lake during smolt sampling operation were obtained from ADF&G and generally span June 1–15. Direct measurements of water temperature were not available for other seasons, but air temperature has been a useful proxy for water temperature [29]. Long-term records of daily air temperature from the Iliamna Airport are available spanning the complete biological time series [42]. To identify potential heterogeneity in rates of change between seasons we considered monthly mean temperatures independently. During several years Iliamna Airport did not report temperature data but data were available from the weather station at Intricate Bay, on the southern shore of Iliamna Lake. Temperatures at the two sites were highly correlated (ρ > 0.95) and combined to complete the time series from 1955–2014. In lakes with seasonal ice coverage the spring thaw effectively initiates the phytoplankton growing season; as such ice out date is an important determinant of growth at higher trophic levels [43]. For Iliamna Lake dates of ice out based on observations from local pilots and other residents are available for most years from 1955–2014. Missing values comprised < 5% of all years and were estimated using linear regression with winter mean low temperature and spring mean high temperature as predictors.

During the marine portion of their life history Bristol Bay sockeye salmon are distributed widely in in the Gulf of Alaska and North Pacific Ocean as they grow to maturity [44]. To account for possible environmental influences on the ocean life history phase we considered indices of large scale climate patterns thought to influence the Bering Sea and North Pacific ecosystems. Following Litzow *et al*. [45] we considered a suite of basin scale indices–notably the Pacific Decadal Oscillation [PDO; 18] and El Nino Southern Oscillation (ENSO)–and regional conditions including sea surface temperature (SST) and surface low pressure [19]. We also included in our trend analysis two regional time series of SST recorded at the Pribilof Islands and at NOAA mooring TsfcM2 over the eastern Bering Sea shelf [46]. Complete source information for all marine environmental variables is provided in S1 Table.

### Statistical analyses

Ordinary least squares (OLS) regression with year as the independent variable was used to test for directional trends in biological and environmental time-series. Data represented as proportions were first square root arcsin transformed to approximate normality. To account for the influence of possible autocorrelation, ρ –the degree of autoregressive first order autocorrelation–was calculated and all relationships were subsequently modeled using a generalized least squares (GLS) regression with a biologically plausible first order autoregressive error structure. The quality of the two models was compared using a likelihood ratio test and coefficients and standard errors from the preferred model were considered more reliable.

Generalized linear and additive models were used to evaluate the strength of these intermediate relationships hypothesized to link growing season temperature and sockeye productivity. For marine survival, a mixed modeling approach was used to account for high interannual variability. In order to minimize the possibility of identifying spurious correlations we restricted our candidate models based on preexisting hypotheses. For each modeled relationship the quality of candidate models was compared using Akaike’s Information Criterion (AIC). Models were further compared by calculating AIC weights which represent the conditional probability of a model relative to all candidate models.

The first intermediate relationship links temperature and density to juvenile growth during lake rearing. Rich and colleagues [29] identified the average mean Iliamna air temperature during March through June as the strongest environmental predictor of fry growth in Iliamna Lake. We utilized OLS regression to evaluate the predictive power of alternative environmental covariates but failed to identify any stronger relationships. No direct estimates of juvenile abundance prior to smolt migration are available. Fry density was therefore indexed as the natural logarithm of the spawning escapement that produced the cohort (e.g. ln(1990 spawners) for 1991 fry) to account for the assumed density dependent reduction in fry production by large escapements. Yearlings–juveniles spawned in the previous year that delay migration until age two–may also compete for food with fry and therefore influence growth. Yearling density was indexed as the natural logarithm of age-2 smolts migrating in the following spring [31]. Because no direct measurements of growth are available, we used average fry length on 1 September as an index of growth in a given year. In order to allow for potential nonlinearity in the relationship between temperature and fry growth we considered generalized additive model (GAM) forms of each linear model in which the coefficient for spring temperature was replaced by an optimal smoothing function fit using penalized regression splines and selected via generalized cross validation [47]. In total we considered ten candidate models (Table 1.)

**Table 1:** Candidate models for intermediate, deterministic relationships.

Response:	ID:	Predictors:	Distribution:
Fry length	FL-1	α + β_1_ * spring temperature + β_2_ * fry + β_3_ * yearlings + ε_t_	Normal
	FL-1gam	α + *f* (spring temperature) + β_1_ *fry + β_2_ * yearlings + εt	
	FL-2	α + β_1_ * spring temperature + β_2_ * (fry + yearlings) + ε_t_	
	FL-2gam	α + *f* (spring temperature) + β1 * (fry + yearlings) + ε_t_	
	FL-3	α + β_1_ * spring temperature + β_2_ * fry + ε_t_	
	FL-3gam	α + *f* (spring temperature) + β1 * fry + ε_t_	
	FL-4	α + β_1_ * spring temperature + β_2_ * yearlings + ε_t_	
	FL-4gam	α + *f* (spring temperature) + β1 * yearlings + ε_t_	
	FL-5	α + β_1_ * spring temperature + ε_t_	
	FL-5gam	α + *f* (spring temperature) + ε_t_	
logit (age-1)	AC-1	α + β_1_ * fry length + β_2_ * spring temp. + β_3_ * year + ε_t_	Beta
	AC-2	α + β_1_ * fry length + β_2_ * condition + β_3_ * year + ε_t_	
	AC-3	α + β_1_ * fry length + β_2_ * year + ε_t_	
	AC-4	α + β_1_ * fry length + β_2_ * spring temperature + ε_t_	
	AC-5	α + β_1_ * fry length + β_2_ condition factor + ε_t_	
	AC-6	α + β_1_ * fry length + ε_t_	
logit (SAS)	SAS-1	α + β_1_ * age + β_2_ * length + ε_t_	Binomial
	SAS-1R	α + *a*_*i*_ + β_1_ * age + β_2_ * length + ε_t_	
	SAS-2	α + β_1_ * age + ε_t_	
	SAS-2R	α + *a*_*i*_ + β_1_ * age + ε_t_	
	SAS-3	α + β_1_ * length + ε_t_	
	SAS-3R	α + *a*_*i*_ + β_1_ * length + ε_t_	
log(R/S)	RS-1	α + β_1_ * SAS + β_2_ * Smolt / Spawner + ε_t_	Log-normal
	RS-2	α + β_1_ * SAS + ε_t_	
	RS-3	α + β_1_ * Smolt / Spawner + ε_t_	
log(R/S)	DC-1	α + β_1_ * escapement + β_2_ * spring temp. + ε_t_	Log-normal

Model ID key: FL = fry length model, AC = age composition model, SAS = marine survival model, RS = freshwater/ marine survival variance partitioning model, DC = direct temperature-productivity correlation model, gam = generalized additive model, R = includes random effects (i.e. mixed model).

The next intermediate relationship links fry growth to age at seaward migration. Because the vast majority of individuals go to sea after either one or two years, migration was considered a binary response variable and therefore required a generalized modelling approach. The data were overdispersed under a binomial model, and we therefore used the more flexible beta distribution–which allows for an independent estimate of variance–with a logit link to model the proportion of smolts migrating after one year. The smolt transformation that precedes seaward migration is complex and includes physiological, morphological and behavioral changes that develop over a period of months [48]. As such, an individual fry must commit to these changes well before the period in spring during which seaward migration occurs or else delay and remain in the lake for another year. This ‘decision’ is apparently based on an evaluation of physiological condition and depends on size, with larger individuals tending to migrate while smaller individuals delay [49]. Because there is little opportunity for additional growth after August in Iliamna Lake, length on 1 September was used as the primary explanatory variable. Initial models showed strong autocorrelation of residuals, indicating that for a given fry length the probability of migrating at age-1 has increased over time and suggesting omission of an important explanatory variable. To achieve independence of residuals we considered several potential explanations for this trend including condition factor (weight at a given length), environmental conditions during the spring of migration, and a linear temporal trend that may capture other unobserved changes. In total we considered six candidate models (Table 1).

We next modeled marine survival as a function of smolt size and age to test the hypothesis that age-2 smolts are more likely to survive and return to breed. As a response variable we used freshwater age-specific marine survival by year of seaward migration (i.e. freshwater age-1 smolts/ freshwater age-1 returns produced by those smolts). Marine survival in sockeye salmon is generally size dependent [50,51] and in the Kvichak River population age-2 smolts are on average 23% longer and 80% heavier than age-1 smolts [23]. However, slower growing individuals are more likely to be age-2 smolts, and age may therefore influence survival independent of length. Additionally, survival in salmon is highly stochastic [51] and as such we anticipated a strong year effect in addition to any size effect. To account for this interannual variability in survival we also considered each model with and without year as a random effect by allowing a unique intercept for each migration year. In total we compared six candidate models (Table 1).

It is in theory possible that variation in total productivity is dominated by fluctuations in survival during the freshwater life history phase and that marine survival is much less variable. To exclude this possibility we modeled the logarithm of recruits per spawner in response to smolt-to-adult survival (S/S) and smolts per spawner SAS. By definition these predictor variables explain 100% of the variation in productivity. However, by standardizing both predictors to zero mean and unit variance the coefficients of the model can be directly compared to evaluate the relative contribution of freshwater and marine factors to total productivity. We also conducted deviance partitioning by dropping either smolts per spawner or SAS from the model and comparing the explained deviance of each. Finally, we evaluated the direct environment-productivity correlation by including spring temperature as a covariate in a linear form of the Ricker stock-recruit model [37], and–to be consistent with the intermediate hypotheses–allowing for a non-linear relationship between temperature and productivity with a smoothing function. All analyses were conducted in R 3.1.3 [52] using packages ‘mgcv’ [47,53], ‘nlme’ [54], ‘betareg’ [55], ‘glmmML’ [56] and ‘lme4’ [57].

### Animal Ethics

This study synthesizes long-term data collected by multiple organizations; no animals were sampled or collected specifically for this study. Data from the Alaska Department of Fish and Game were retrieved from publicly available sources. Data provided by the Fisheries Research Institute were collected in accordance with the IACUC protocols prescribed by the University of Washington’s office of animal welfare and with approval from the Alaska Department of Fish and Game. No threatened or endangered species were involved in this study.

## Results

### Trend analysis

Significant directional trends were identified in many environmental and biological time series. However, in general even statistically significant trends explained little of the total variation in the time series (maximum R^2^ = 0.26). For freshwater environmental variables all slopes were consistent with a general warming trend. Nine of twelve trends in monthly mean temperatures were significant at α = 0.1. June Kvichak River water temperature and Iliamna Lake ice breakup date showed significant positive and negative trends respectively, neither with significant autocorrelation. Complete results for trend analysis of freshwater environmental variables are provided in Table 2 and selected trends are shown in S1 Fig. For the marine environmental variables both the direction and magnitude of temporal trends varied. Three of ten variables displayed directional trends at a significance level of α = 0.1 and eight of ten time series were significantly autocorrelated. Both the annual and winter PDO indices displayed marginally significant positive trends (p = 0.068 and 0.032 respectively). Of the three time series of regional SST only one–southeastern Bering Sea spring temperature (May SST)–showed a directional trend; in this case cooling (p = 0.004). Complete results for trend analysis of marine environmental variables are provided in Table 3 and trend for May SST is shown in S1 Fig.

**Table 2:** Summary of trend analysis results for freshwater environmental variables.

	slope	R^2^	p-value	AR (1) slope	ρ	LRT p-value
**Iliamna Airport air temperatures (°C):**						
January mean	*0*.*006 (-0*.*009*,*0*.*021)*	*0*.*01*	*0*.*438*	0.006 (-0.012,0.024)	0.15	0.14
February mean	*0*.*016 (0*.*001*,*0*.*03)*	*0*.*08*	***0*.*033***	0.016 (0.001,0.031)	0.05	0.538
March mean	*0*.*007 (-0*.*008*,*0*.*022)*	*0*.*01*	*0*.*379*	0.007 (-0.011,0.024)	0.12	0.231
April mean	0.021 (0.007,0.035)	0.13	**0.004**	*0*.*022 (0*.*004*,*0*.*04)*	*0*.*20*	**0.062**
May mean	0.026 (0.013,0.039)	0.21	**< 0.001**	*0*.*027 (0*.*01*,*0*.*045)*	*0*.*23*	**0.035**
June mean	0.024 (0.01,0.038)	0.17	**0.001**	*0*.*024 (0*.*007*,*0*.*042)*	*0*.*20*	**0.061**
July mean	0.022 (0.008,0.036)	0.14	**0.003**	*0*.*022 (0*.*005*,*0*.*04)*	*0*.*20*	**0.065**
August mean	*0*.*020 (0*.*006*,*0*.*034)*	*0*.*12*	***0*.*007***	0.020 (0.004,0.037)	0.15	0.147
September mean	*0*.*013 (-0*.*002*,*0*.*028)*	*0*.*05*	***0*.*079***	0.013 (0.001,0.025)	-0.20	0.201
October mean	*0*.*015 (0*.*001*,*0*.*03)*	*0*.*07*	***0*.*039***	0.015 (0.004,0.027)	-0.21	0.155
November mean	*0*.*003 (-0*.*012*,*0*.*018)*	*0*.*00*	*0*.*67*	0.003 (-0.012,0.018)	-0.03	0.962
December mean	*0*.*019 (0*.*004*,*0*.*033)*	*0*.*10*	***0*.*012***	0.018 (0.005,0.032)	-0.07	0.776
Spring (Mar.-Jun.) mean	0.020 (0.006,0.034)	0.12	**0.006**	*0*.*021 (0*.*001*,*0*.*041)*	*0*.*32*	**0.005**
Autumn (Sep.—Nov.) mean	*0*.*008 (-0*.*007*,*0*.*023)*	*0*.*02*	*0*.*307*	0.008 (-0.007,0.023)	-0.01	0.852
Spawning season mean high	0.010 (-0.005,0.025)	0.03	0.174	*0*.*012 (-0*.*011*,*0*.*035)*	*0*.*39*	**0.001**
Growing season degree days	0.030 (0.017,0.042)	0.26	**< 0.001**	*0*.*032 (0*.*011*,*0*.*053)*	*0*.*41*	**< 0.001**
Winter mean low	*0*.*019 (0*.*005*,*0*.*033)*	*0*.*11*	***0*.*01***	0.021 (0.002,0.04)	0.25	**0.022**
H_2_O temp. at smolt migration	*0*.*014 (0*,*0*.*029)*	*0*.*06*	***0*.*056***	0.014 (-0.002,0.031)	0.10	0.302
Ice breakup (Day of Year)	*-0*.*022 (-0*.*036*,*-0*.*008)*	*0*.*14*	***0*.*003***	-0.022 (-0.038,-0.005)	0.14	0.178

OLS = Ordinary least squares models. GLS = Generalized Least Squares models with first order autocorrelation. LRT = Likelihood ratio test between OLS and GLS models. Bold indicates significance at α = 0.10, italics indicate preferred model based on likelihood ratio test.

**Table 3:** Summary of trend analysis results for marine environmental variables.

	slope	R2	p-value	AR (1) slope	ρ	LRT p-value
PDOa	0.013 (-0.001,0.026)	0.05	**0.0681**	*0*.*012 (-0*.*015*,*0*.*038)*	*0*.*56*	**<0.001**
NPI	*-0*.*008 (-0*.*022*,*0*.*005)*	*0*.*02*	*0*.*2282*	-0.008 (-0.023,0.006)	0.02	0.6648
PDOw	0.015 (0.001,0.028)	0.07	**0.0321**	*0*.*015 (-0*.*003*,*0*.*032)*	*0*.*23*	**0.0342**
ENSO	0.007 (-0.007,0.021)	0.02	0.2910	*0*.*008 (-0*.*013*,*0*.*028)*	*0*.*35*	**0.0016**
AO	0.01 (-0.003,0.024)	0.04	0.1331	*0*.*01 (-0*.*007*,*0*.*027)*	*0*.*18*	**0.0958**
NPGO	0.015 (0.001,0.028)	0.07	**0.0332**	*0*.*016 (-0*.*011*,*0*.*044)*	*0*.*59*	**<0.001**
PNA	*0*.*008 (-0*.*006*,*0*.*022)*	*0*.*02*	*0*.*2630*	0.008 (-0.005,0.02)	-0.11	0.5278
MaySST	-0.024 (-0.036,-0.011)	0.19	**0.0004**	*-0*.*022 (-0*.*044*,*0)*	*0*.*45*	**<0.001**
STM2	-0.009 (-0.023,0.005)	0.03	0.2130	*-0*.*009 (-0*.*027*,*0*.*009)*	*0*.*25*	**0.0236**
PISST	0.009 (-0.005,0.023)	0.03	0.1925	*0*.*009 (-0*.*01*,*0*.*029)*	*0*.*29*	**0.0081**

OLS = Ordinary least squares models. GLS = Generalized Least Squares models with first order autocorrelation. LRT = Likelihood ratio test. Bold indicates significance at α = 0.10, italics indicate preferred model based on likelihood ratio test.

In total eight of 25 biological variables trended significantly over the period of observation at a significance level of α = 0.1. Changes were not uniformly distributed across variable categories. Of 14 abundance and productivity variables only one–age 1.3 abundance–changed over time with a significant positive trend (p = 0.048). On the other hand, all three age composition variables displayed directional trends with proportions of freshwater age-1 increasing and marine age-2 decreasing. Three of six freshwater growth indices indicated directional change with negative trends in length for both age-1 and age-2 and increasing condition factor for age-1 smolts. Finally, the only phenological variable–median smolt migration date–showed a declining trend (i.e., earlier migration: p = 0.007). Growth variables were generally autocorrelated at a one year lag with ρ values between 0.12 and 0.36, whereas abundance variables were not. However, of all biological variables the R/S index displayed the strongest autocorrelation with a ρ of 0.50. Complete results for trend analysis of biological variables are provided in Table 4 and selected trends are shown in S2 Fig.

**Table 4:** Summary of trend analysis results biological variables.

Variable	OLS Slope (95% C.I.)	R^2^	p-value	GLS Slope (95% C.I.)	ρ	LRT p-value
**Abundance:**						
Run Size	*-0*.*131 (-0*.*302*,*0*.*041)*	*0*.*04*	*0*.*133*	-0.132 (-0.329,0.065)	0.12	0.239
Escapement	*-0*.*057 (-0*.*132*,*0*.*019)*	*0*.*04*	*0*.*137*	-0.057 (-0.133,0.02)	0.01	0.758
Age 1.2	*-0*.*013 (-0*.*083*,*0*.*058)*	*0*.*00*	*0*.*725*	-0.013 (-0.079,0.054)	-0.07	0.777
Age 2.2	*-0*.*103 (-0*.*259*,*0*.*053)*	*0*.*03*	*0*.*192*	-0.102 (-0.261,0.058)	0.01	0.756
Age 1.3	*0*.*019 (0*,*0*.*037)*	*0*.*07*	***0*.*048***	0.019 (-0.001,0.038)	0.05	0.542
Age 2.3	*-0*.*002 (-0*.*02*,*0*.*017)*	*0*.*00*	*0*.*850*	-0.002 (-0.021,0.018)	0.04	0.573
Total return	*-0*.*112 (-0*.*329*,*0*.*106)*	*0*.*02*	*0*.*309*	-0.111 (-0.327,0.104)	-0.02	0.928
Smolt abundance	*-1*.*141 (-3*.*18*,*0*.*898)*	*0*.*03*	*0*.*266*	-1.141 (-3.135,0.853)	-0.03	0.986
Age-1 smolt abund.	*-0*.*128 (-1*.*041*,*0*.*785)*	*0*.*00*	*0*.*779*	-0.122 (-1.183,0.938)	0.15	0.189
Age-2 smolt abund.	*-1*.*095 (-2*.*473*,*0*.*283)*	*0*.*05*	*0*.*117*	-1.093 (-2.466,0.281)	-0.01	0.874
**Productivity:**						
R/S	0.000 (-0.034,0.033)	0.00	0.992	*-0*.*003 (-0*.*055*,*0*.*048)*	*0*.*39*	**0.001**
R/S Index	0.004 (-0.011,0.019)	0.01	0.604	*0*.*002 (-0*.*025*,*0*.*028)*	*0*.*50*	**<0.001**
Smolt per spawner	*-0*.*150 (-0*.*394*,*0*.*095)*	*0*.*03*	*0*.*224*	-0.158 (-0.436,0.121)	0.13	0.253
SAS	*0*.*001 (-0*.*001*,*0*.*002)*	*0*.*02*	*0*.*350*	0.001 (-0.001,0.003)	0.10	0.206
**Age Composition:**						
% FW age-2 (return)	*0*.*006 (0*.*003*,*0*.*009)*	*0*.*22*	***<0*.*001***	0.006 (0.003,0.01)	0.10	0.309
% Ocean age-2 (return)	*-0*.*003 (-0*.*005*,*-0*.*001)*	*0*.*16*	***0*.*002***	-0.003 (-0.005,-0.001)	-0.15	0.385
% Age-1 smolts (brood)	*0*.*003 (0*,*0*.*006)*	*0*.*07*	***0*.*064***	0.003 (0,0.006)	0.01	0.696
**Freshwater Growth:**						
Age-1 smolt length	-0.088 (-0.15,-0.027)	0.13	**0.006**	*-0*.*086 (-0*.*169*,*-0*.*003)*	*0*.*26*	**0.019**
Age-2 smolt length	-0.098 (-0.196,0)	0.07	**0.049**	*-0*.*098 (-0*.*231*,*0*.*036)*	*0*.*24*	**0.023**
Age-1 smolt weight	-0.011 (-0.026,0.004)	0.04	0.144	*-0*.*010 (-0*.*03*,*0*.*01)*	*0*.*27*	**0.013**
Age-2 smolt weight	-0.025 (-0.056,0.006)	0.05	0.110	*-0*.*024 (-0*.*072*,*0*.*023)*	*0*.*29*	**0.002**
Age-1 condition factor	0.002 (0.001,0.003)	0.18	**0.001**	*0*.*002 (0*,*0*.*003)*	*0*.*35*	**0.0028**
Age-2 condition factor	0.000 (-0.001,0.001)	0.00	0.720	*0*.*00 (-0*.*001*,*0*.*002)*	*0*.*36*	**0.002**
Fry length	*0*.*023 (-0*.*09*,*0*.*135)*	*0*.*00*	*0*.*687*	0.033 (-0.107,0.172)	0.12	0.101
**Phenology:**						
50% smolt migration	*-0*.*140 (-0*.*24*,*-0*.*04)*	*0*.*14*	***0*.*007***	-0.139 (-0.241,-0.037)	0.02	0.764

OLS = Ordinary least squares models. GLS = Generalized Least Squares models with first order autocorrelation. LRT = Likelihood ratio test. Bold indicates significance at α = 0.10; italics indicate preferred model based on likelihood ratio test.

### Intermediate relationship models

**Fig 4:**
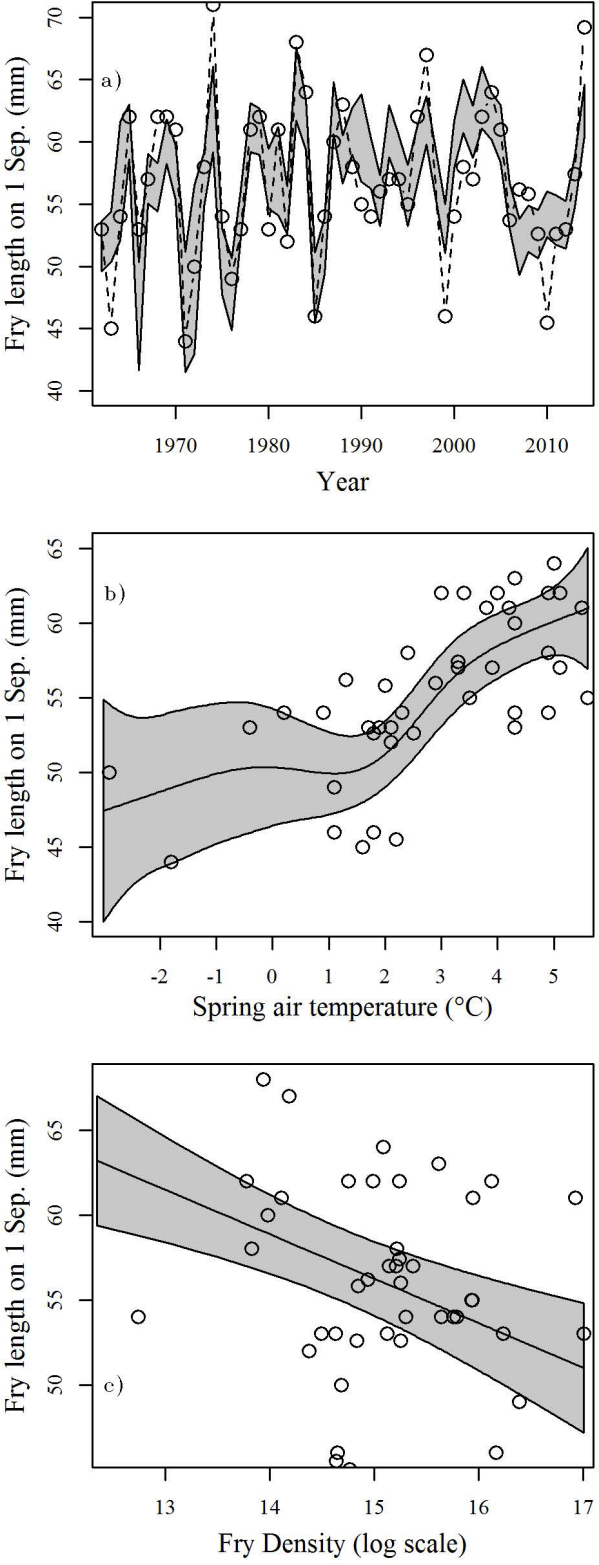
Results from AIC selected fry length model. a) Observed (points and dashed lines) and predicted (shaded area: 95% confidence interval) fry length. Partial plots showing effects of b) March-June mean air temperature and, c) sockeye fry density on growth during freshwater rearing.

For the relationship between environmental conditions and fry growth the AIC selection process favored a model which included a positive and slightly nonlinear effect of March-June average air temperature (i.e., bigger fish with warmer conditions: estimated DF = 4.012) and a negative, linear effect of fry density (FL-3gam). Several other models in which these two predictors were also included received smaller, but non-zero AIC weights. The selected model received a weight of 0.52. Although a model in which yearling density was added as a predictor (FL-1gam) explained slightly more of the total deviance, it also had a marginally higher AIC and received a weight of only 0.33. The highest ranked model explained over 62% of total deviance (Fig 4A). Across all candidate models spring temperature was a highly significant predictor (p < 0.001). When included, fry density was always highly significant (p < 0.003) whereas yearling density was never significant (p > 0.208). For the preferred model the effect of temperature was essentially flat below 1.5°C, and nearly linear above with fry length increasing ~4.5 mm per°C (Fig 4B). The effect of density was negative with fry length decreasing ~ 2.6 mm per order of magnitude of adults in the parental generation (Fig 4C).

**Fig 5:**
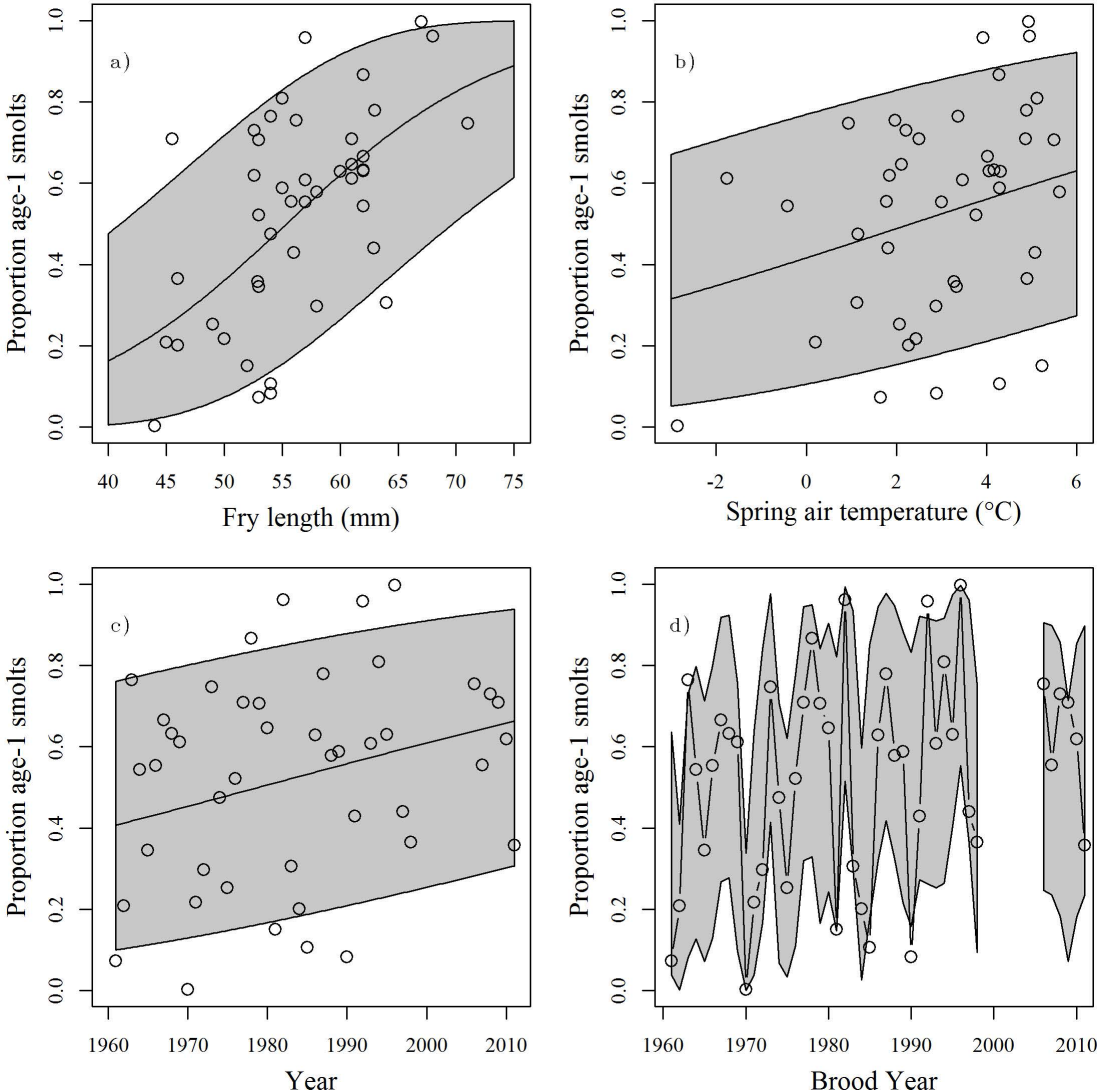
Results from AIC selected smolt age composition model. Partial plots showing the effects of a) fry length, b) spring air temperature in year of migration and c) year of migration on proportion of a brood year migrating at age-1.d) Observed (points and lines) and predicted (shaded area: 95% confidence interval) proportions.

The AIC-preferred smolt age composition model linking freshwater growth and age at seaward migration included fry length on 1 September (Fig 5A), March-June average temperature in year of migration (Fig 5B) and a linear temporal trend (Fig 5C) as predictor variables. The preferred model (AC-1) had the lowest AIC score and received a 0.55 AIC weight. Most of the remaining AIC weight went to models which were nested within the selected model and dropped either the temperature or temporal predictor. The highest ranked model explained over 72% of total deviance (Fig 5D). Fry length was included in all models and was always a highly significant predictor of smolt age (p < 0.001). Age-1 smolt condition factor was not included in the selected model and was not significant in any candidate model (p > 0.228). Where included, both year and spring temperature were always significant (p < 0.023 and p < 0.045 respectively). For AC-1 the effects of fry length and spring temperature on the probability of migrating at age-1 were both positive; the odds of migration increased at ~12% per mm and ~14% per°C, respectively. After accounting for the effects of these other predictors an increasing temporal trend of ~2% per year in the odds of age-1 migration remained significant.

**Fig 6:**
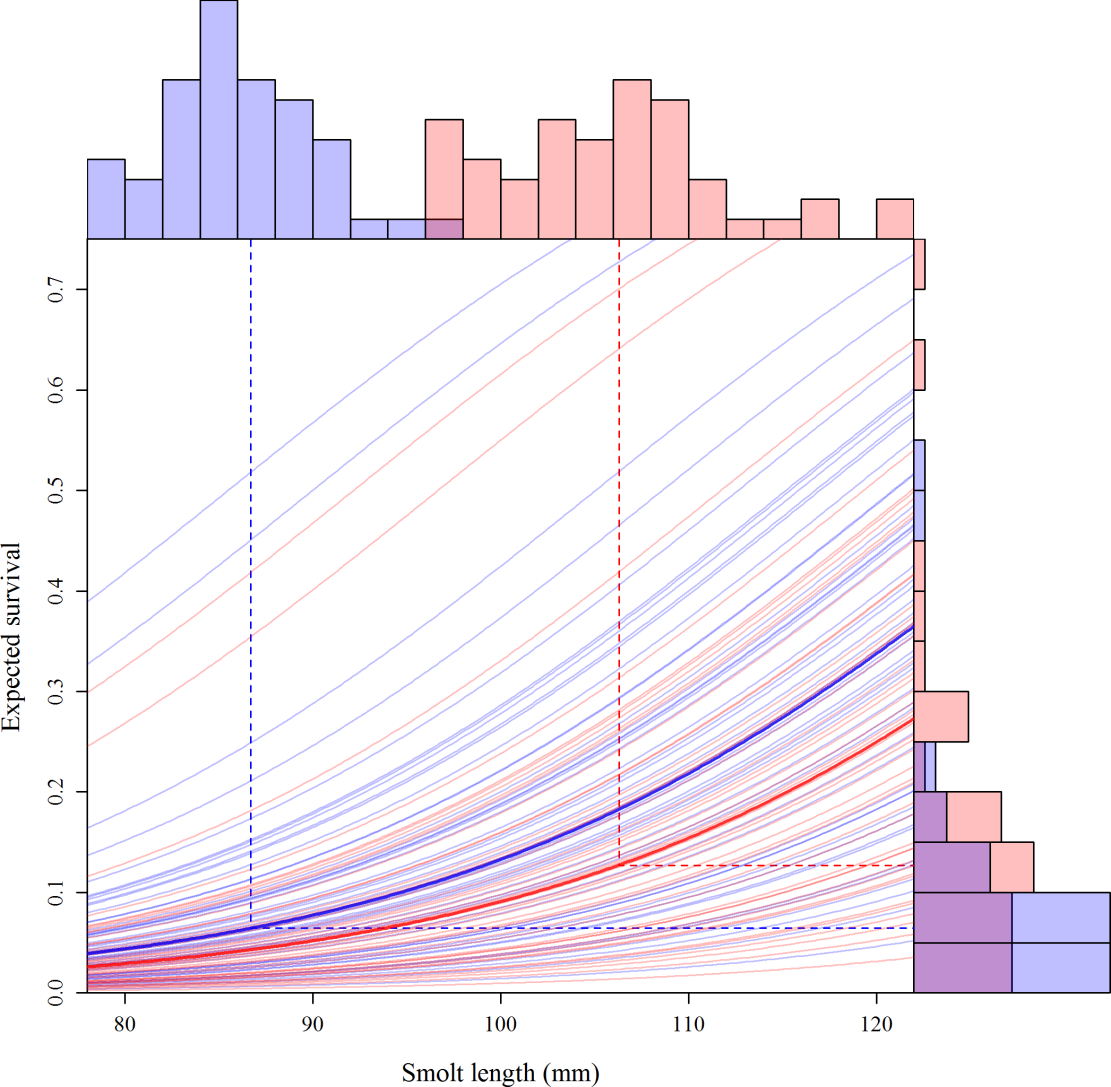
Results from AIC selected smolt survival model. Expected marine survival is shown in relation to length for age-1 (blue) and age-2 (red) smolts. Light colored lines show predicted survival as a function of length for each age in each year (random intercepts from model SAS-1R). Bold lines indicate mean values across all years. Histograms show frequency distributions of smolt length (x-axis) and expected survival at mean length (y-axis) for age-1 and age-2 smolts; dashed lines indicate medians of each frequency distribution, for each smolt age.

For the smolt survival models the AIC selection process strongly favored SAS-1R which included smolt length and age as fixed effects and migration year as a random effect. No other model received any AIC weight and the selected model explained over 86% of total deviance. However, the large majority of deviance explained is attributable to the random intercepts of migration year; model SAS-1 had the same fixed effects, but no random effects and explained only 9.4% of total deviance. The fixed effects of age and length were highly significant (p < 0.001) in all cases where included in a candidate model. In SAS-1R –the highest ranked model–the odds of survival increase ~6% per mm of smolt length. For a given length the effect of age is negative with the odds of survival for age-2 smolts 30% lower than age-1. The effect of the random intercept by year was very large with expected probability of survival for a smolt of average length ranging from 0.006 to 0.518 for age-1 smolt and 0.014 to 0.70 for age-2 smolt (Fig 6). Full results of the model comparisons are provided in Table 5.

**Table 5:** Summary of intermediate relationship model results.

ID	Coefficients (p-values)				AIC	AIC weight	% Dev. Explained
	Spring temp.	Fry	Yearlings				
FL-1	2.01 (**<0.001**)	-2.27 (**0.002**)	-0.05 (0.328)		267.3	0.03	53.6
FL-1gam	NA (**<0.001**)	-2.52(**<0.001**)	-0.05 (0.452)		262.8	0.33	63.1
FL-2	1.88 (**<0.001**)	-1.10 (**0.006**)			269.6	0.01	49.0
FL-2gam	NA (**<0.001**)	-1.15 (**0.003**)			267.0	0.04	57.2
FL-3	2.08 (**<0.001**)	-2.36 (**0.002**)	—		266.3	0.06	52.5
FL-3gam	NA (**<0.001**)	-2.61(**<0.001**)	—		261.9	0.52	62.3
FL-4	2.38 (**<0.001**)	—	-0.68 (0.208)		276.0	0.00	41.1
FL-4gam	NA (**<0.001**)	—	-0.69 (0.452)		274.3	0.00	49.7
FL-5	2.02 **(<0.001**)	—	—		275.7	0.00	38.8
FL-5gam	NA (**<0.001**)	—	—		274.3	0.00	47.7
	**Fry length**	**Spring temp.**	**Cond. Factor**	**Year**			
AC-1	0.106 (**<0.001**)	0.146 (**0.045**)	—	0.021 (**0.023**)	-24.2	0.55	72.1
AC-2	0.119 (**<0.001**)	—	0.109 (0.961)	0.026 (**<0.001**)	-20.2	0.07	67.0
AC-3	0.100 (**<0.001**)	—	—	0.026 (**<0.001**)	-22.2	0.20	66.9
AC-4	0.119 (**<0.001**)	0.187 (**0.011**)	—	—	-21.5	0.14	68.7
AC-5	0.114 (**<0.001**)	—	2.55 (0.228)	—	-16.2	0.01	61.5
AC-6	0.116 (**<0.001**)	—		—	-16.8	0.01	59.1
	**Length**	**Age**	**Year (var.)**				
SAS-1	-0.018 (**<0.001**)	0.775 **(<0.001)**	—		2.9 · 10^8^	0.00	9.4
SAS-1R	0.060 (**<0.001**)	-0.402 (**<0.001**)	1.023		4.4 · 10^7^	1.00	86.6
SAS-2	—	0.503 (**<0.001**)	—		3.0 · 10^8^	0.00	8.6
SAS-2R	—	0.745 (<**0.001**)	0.931		4.8 · 10^7^	0.00	85.3
SAS-3	0.02 (**<0.001**)	—	—		3.2 · 10^8^	0.00	4.4
SAS-3R	0.040 (**<0.001**)	—	0.977		4.5 · 10^7^	0.00	86.4
	**SAS**	**Smolt/Spawner**					
RS-1	0.702 (**<0.001**)	0.609 (**<0.001**)			NA	NA	100
RS-2	0.569 (**<0.001**)	—			NA	NA	46.3
RS-3	—	0.464 (**<0.001**)			NA	NA	32.6
	**Escapement**	**Spring temp.**					
DC-1	-0.011 (0.621)	NA (0.533)			NA	NA	4.12

Best fit model values are underlined. Model ID key: FL = fry length model, AC = age composition model, SAS = marine survival model, RS = freshwater/ marine survival variance partitioning model, DC = direct temperature-productivity correlation model, gam = generalized additive model, R = includes random effects (i.e. mixed model).

For the remaining two hypothesized relationships we modeled productivity in terms of recruits per spawner, first evaluating the relative influence of freshwater and marine life-history phases, and then directly evaluating the effect of temperature during freshwater rearing. Because both predictors were standardized prior to analysis, the coefficients for SAS and smolts per spawner in model RS-1 represent the change in R/S in response to a one standard deviation increase in the predictor. Not surprisingly, both predictors were highly significant (p < 0.001), but direct comparison of the coefficients revealed a ~16% larger response in productivity to SAS than S/S. Deviance partitioning of the model further supported this result as SAS individually explained 46.3% of total deviance while S/S explained only 32.6%. Finally, incorporation of spring temperature as a covariate in the linear Ricker model revealed a slightly convex, but non-significant environmental effect (S3 Fig). Thus, no direct correlation between temperature during freshwater rearing and overall population productivity was apparent.

## Discussion

Identifying and accurately attributing biological responses to climate change is, and will continue to be, vital for conservation and management of animal populations in a warming world. This field of research will inform important policy decisions and as such, requires a particularly thoughtful and deliberate approach. Because of the challenges of conducting large-scale ecological experiments, correlative approaches will be required. Here we utilized a multi-step approach with a series of intermediate mechanistic hypotheses to address the shortcomings of a purely correlative approach between climate and productivity. We also contextualized the productivity-environment relationship by examining trends in potentially confounding environmental and biological variables. Taken together, these analyses allow for robust conclusions regarding the importance of freshwater climate in determining Kvichak sockeye productivity; by extension, they also provide lessons for climate effects on other species with complex life histories and migratory cycles.

Considering first the intermediate, deterministic relationships that link temperature during freshwater rearing to population productivity, there appears to be strong support for a link between freshwater climate and productivity. As predicted, freshwater growth as measured by fry size on 1 September was positively related to average March-June air temperature and negatively related to density; these two variables explained over 60% of deviance in fry growth. This finding is consistent with other studies of sockeye salmon growth though the relative importance of temperature and density vary among systems [58,59]. Also as predicted, and consistent with previous research on Kvichak River sockeye [29,30], the probability of seaward migration at age-1 was positively correlated with fry size, which alone explained over 50% of the deviance in migration probability. We also identified an unexpected increasing propensity over time for age-1 migration that was independent of size. Spring temperature in the year of migration may partially explain this phenomenon, but a linear temporal trend capturing other, unknown processes was also significant. Possible explanations for this phenomenon include evolution and shifting stock composition of the Kvicahk sockeye run [25]. It is possible that this trend indicates evolution of the reaction norm between size and probability of age-1 seaward migration [60]. Maturation reaction norms respond to selection in fishes, and a shift in the relative lifetime survival of the two smolt ages could provide the selection necessary in this case [61]. Alternatively, this trend may be explained by a change in the relative contribution of different populations within the Kvichak basin to system’s total productivity. Although abundance data are available only at the basin level, many distinct populations make up the total Kvichak run and population-specific propensities for freshwater age have been documented [30].

Marine survival was positively and significantly related to smolt size; the larger age-2 smolts experienced on average 95% higher survival than age-1 smolts at their respective median lengths. This magnitude of size selective smolt mortality is consistent with patterns observed in sockeye salmon throughout their range [50] as well as with previous studies in the Kvichak River system [23,27]. Finally, marine survival accounted for the majority of variation in population productivity. However, although all the intermediate deterministic relationships were statistically significant with reasonably strong explanatory power, the predicted negative correlation between growing season temperature and overall productivity was not. Indeed, based on the correlative approach spring temperature appears to have had little or no influence on population productivity of Kvichak River sockeye salmon.

The trend analyses can provide some insight into this disconnect between the significant deterministic intermediate relationships and lack of an overall influence of spring temperature on productivity. In the freshwater environments there is strong evidence of a long-term warming trend and environmental indices previously linked to juvenile sockeye salmon growth and phenology including spring air temperature, ice out date and water temperature during smolt migration each display directional change over the period of record [62–64]. Despite this, freshwater growth has not displayed the increasing trend that might be expected under warming conditions. Mean fry length on 1 September has remained stable over the past five decades despite inter-annual variation > 25 mm. Meanwhile–and somewhat counterintuitively–mean length of both age-1 and age-2 smolts has declined. This has likely resulted from a combination of factors. First, timing of smolt migration has advanced by over a week since 1955, and earlier migrating fish forego some growth opportunity in the lake. Second, this trend could result from shifting smolt age composition. Individual fry near the margin of migrating at age-1 and age-2 will fall near the low end of the age-1 length distribution, but at the high end of the age-2 growth distribution. Thus, if more of these individuals migrate at age-1 there are more relatively small age-1 smolts and fewer faster growing, larger age-2 smolts. The trend analysis indicates a significant shift toward lower smolt age which is therefore consistent with the observed patterns in mean smolt length. Indeed, age composition showed the most significant changes over time of all biological variables. Kvichak sockeye are on average migrating to sea at a younger age and spending longer at sea as indicated by increasing proportions of freshwater age-1 (rather than 2) and marine age-3 (rather than 2) individuals. Despite these shifts and the observed ~95% marine survival advantage of age-2 smolts overall smolt-to-adult survival has not declined. This apparent paradox is key to understanding the disagreement between the mechanistic and correlative approaches

Mixed effects modeling of smolt-to-adult survival allows for examination of the relative importance of the fixed effects of smolt size and age, and random interannual variation in marine survival. Consistent with the meta-analysis of sockeye marine survival conducted by Koenings and colleagues [50], our analysis revealed that although both age and length were significant predictors of marine survival, they together explained little of the total variability. Similarly, Henderson and Cass [51] found that in Chilko Lake sockeye marine SAS was positively correlated with length within a cohort, but length was not a significant predictor of survival over a 34 year time series. As such, although the intermediate hypotheses demonstrate a mechanism by which declining smolt age should reduce average marine survival, exogenous factors dominate the signal of variability in marine survival and effectively break the chain of relationships that link spring temperatures during freshwater growth to population productivity. Additionally, our analysis necessarily ignored differential freshwater survival of the two smolt ages because freshwater survival and the proportion of a brood year migrating at age-1 are confounded. Our estimates of differential SAS therefore represent an upper bound on the lifetime survival advantage of age-2 smolts. In reality, some portion of this advantage is offset by the risk of mortality associated with a second year of lake residence; though this risk is thought to be low relative to the marine environment [22]. Therefore, contrary to our prediction, any potential negative influence of increased freshwater growth on population productivity is either compensated for by reduced freshwater mortality in age-1 smolts or unobservable in our time-series as a result of the stochastic nature of marine survival. Despite this, the results of testing the intermediate hypotheses clearly show that changes in temperature during freshwater rearing have profound consequences for growth, life history and demographics in Kvichak River sockeye. Observed warming over the past six decades has shifted the dominant life-history strategy towards earlier seaward migration which in turn has altered the average length of marine residence. Together, these changes impact generation time, size at maturity and the relative importance of the marine and freshwater environments in determining lifetime survival. Thus, relying solely on correlation with productivity for identifying the influences of climate change on Kvichak River sockeye would have overlooked important changes with implications for the biology and management of the stock.

In addition to demonstrating the shortcomings of a purely correlative approach to studying climate change impacts on productivity, our analyses also highlight heterogeneity in patterns of environmental change. While in the freshwater environment there has been a clear warming trend, the rate of change differed among seasons, with spring and summer temperatures increasing most markedly. Associated with rising spring air temperatures, the typical date of ice out has advanced by nearly two weeks, resulting in a longer ice free growing season in Iliamna Lake. In addition to controlling growth opportunity through primary production, ice out also largely determines when water temperatures begin to increase in spring. Both ice out date and water temperature modulate the seasonal timing of smolt migration [22,65], and as such warming is likely responsible for the observed trends in smolt size and median migration date discussed previously. In other salmon populations, early marine survival may depend on the degree of synchrony between ocean entry date and favorable environmental conditions [66]. Smolts can only respond to freshwater environmental cues to initiate migration [67], and matching migration timing with the period of optimal survival is therefore mediated by the long term relationship between freshwater and marine environments. Thus, our observation of declining spring SST in the Eastern Bering Sea is particularly interesting because it indicates that optimal marine conditions may be occurring later while freshwater environmental cues are initiating migration earlier. Further research in this area may help to explain some of the large year effect identified by our model of marine survival.

Taken together, our results describe a salmon population complex that has experienced directional environmental change that is both spatially and temporally heterogeneous and displayed multiple, simultaneous biological responses. This type of complexity is likely to be the rule rather than the exception for animal populations faced with environmental change. Because of this complexity, even when robust, deterministic relationships link environmental conditions to survival climate change impacts on productivity may remain difficult to detect using strictly correlative approaches. Previous studies have highlighted the issue of false positives in the search for environmental drivers of biological change [7,10]; our results demonstrate the reciprocal issue of false negatives. If, instead of working from an *a priori* hypothesis we explored correlations between Kvichak River sockeye salmon productivity and a broad range of environmental covariates, spring temperature during freshwater rearing would have been dropped as non-significant. By instead considering the explicit mechanisms by which environmental drivers elicit biological responses, we revealed that although a direct correlation with productivity is not apparent, climate warming has precipitated other important biological changes in the world’s largest sockeye salmon stock complex.

Although our analyses were facilitated by an unusually rich data set with large sample sizes at multiple life history stages over many decades, the implications of our results are broadly applicable to the study of biological responses to climate change. Identifying and predicting climate impacts on population productivity is vital for natural resource management and the conservation of rare or threatened species. While testing for significance of environment-productivity relationships is a fast and relatively low cost way of detecting potential climate change impacts, type 1 and type 2 errors are both major concerns. Stating and testing the mechanisms that link environment and species can improve the method by helping to simultaneously identify significant, but spurious and biologically important, but non-significant correlations. Even in the absence of extensive data on life-history, phenology or physiology, logical consideration of the mechanisms that link environmental and biological change is possible. Moving beyond correlation can inform more robust conclusions from existing data and help ensure that future research is targeted toward the most relevant questions as climate change continues to impact populations and ecosystems.

## Supporting Information

S1 Data Biological data for brood years 1954–2014. (CSV)

S2 Data Environmental data for 1955–2014. (CSV)

S1 Fig Temporal trends in environmental variables. a) March-June spring air temperature at Iliamna Airport, b) day of year on which ice breakup on Iliamna Lake is complete, c) mean May sea surface temperature in the eastern Bering Sea. Lines indicate GCV-selected best fit penalized regression spline; shaded areas show 95% confidence intervals and are included to highlight general trends.
(TIF)

S2 Fig Temporal trends sockeye salmon biological variables. a) Proportion of brood year returning as freshwater age-1, b) proportion of brood year returning as ocean age-3, c) condition factor of age-1 sockeye smolts, d) date on which 50% of total smolt migration is reached. Lines indicate GCV-selected best fit penalized regression spline; shaded areas show 95% confidence intervals and are included to highlight general trends.
(TIF)

S3 Fig No significant direct correlation between temperature and productivity. Partial plot of the influence of spring air temperature during freshwater rearing on productivity (R/S). Points show observed data, lines indicates GCV-selected best fit penalized regression spline; shaded area show 95% confidence interval.
(TIF)

S1 Table Description and sources of all marine environmental variables. (XLSX)
