# Supplementary figures and images for: Beyond Correlation in the Detection of Climate Change Impacts: Testing a Mechanistic Hypothesis for Climatic Influence on Sockeye Salmon (Oncorhynchus nerka) Productivity
Source: PLoS One. 2016 Apr 28;11(4):e0154356. doi: 10.1371/journal.pone.0154356 (PMC4849749; doi:10.1371/journal.pone.0154356)

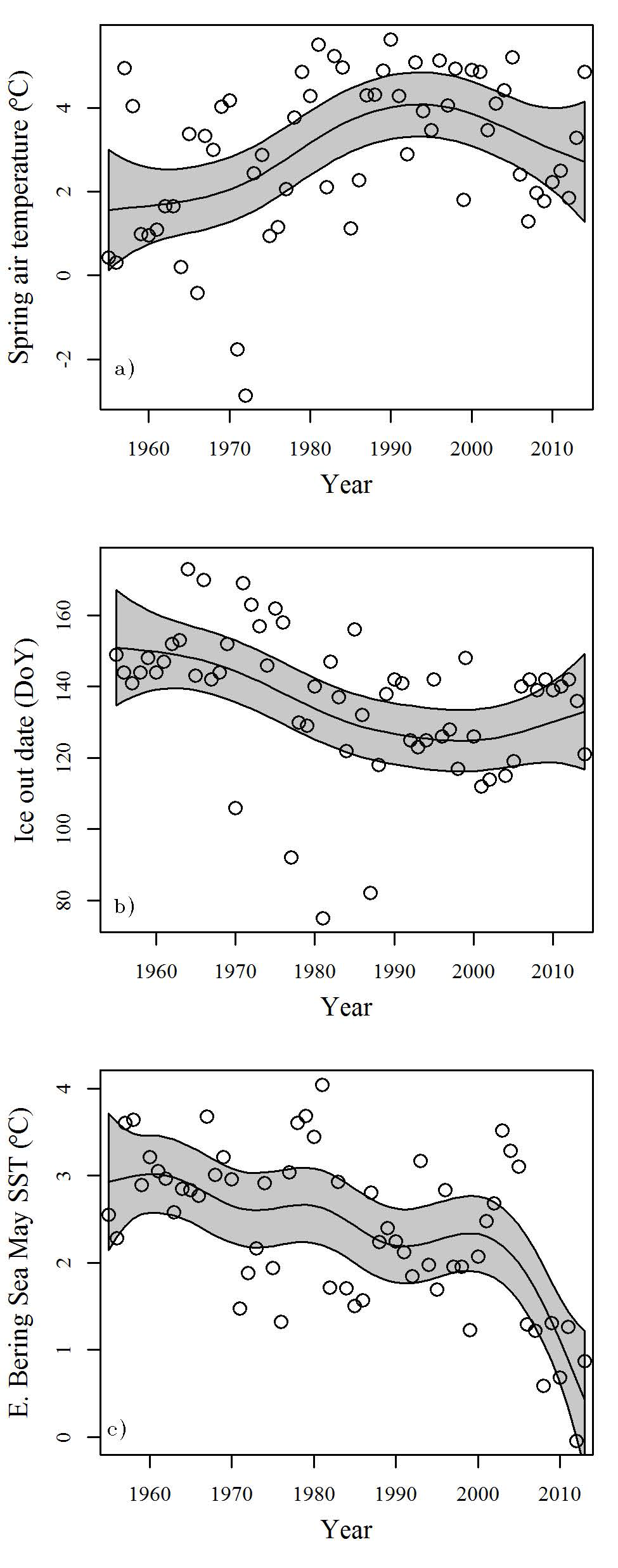

Supplement: S1 Fig — a) March-June spring air temperature at Iliamna Airport, b) day of year on which ice breakup on Iliamna Lake is complete, c) mean May sea surface temperature in the eastern Bering Sea. Lines indicate GCV-selected best fit penalized regression spline; shaded areas show 95% confidence intervals and are included to highlight general trends. (TIF) [file pone.0154356.s003.tif]

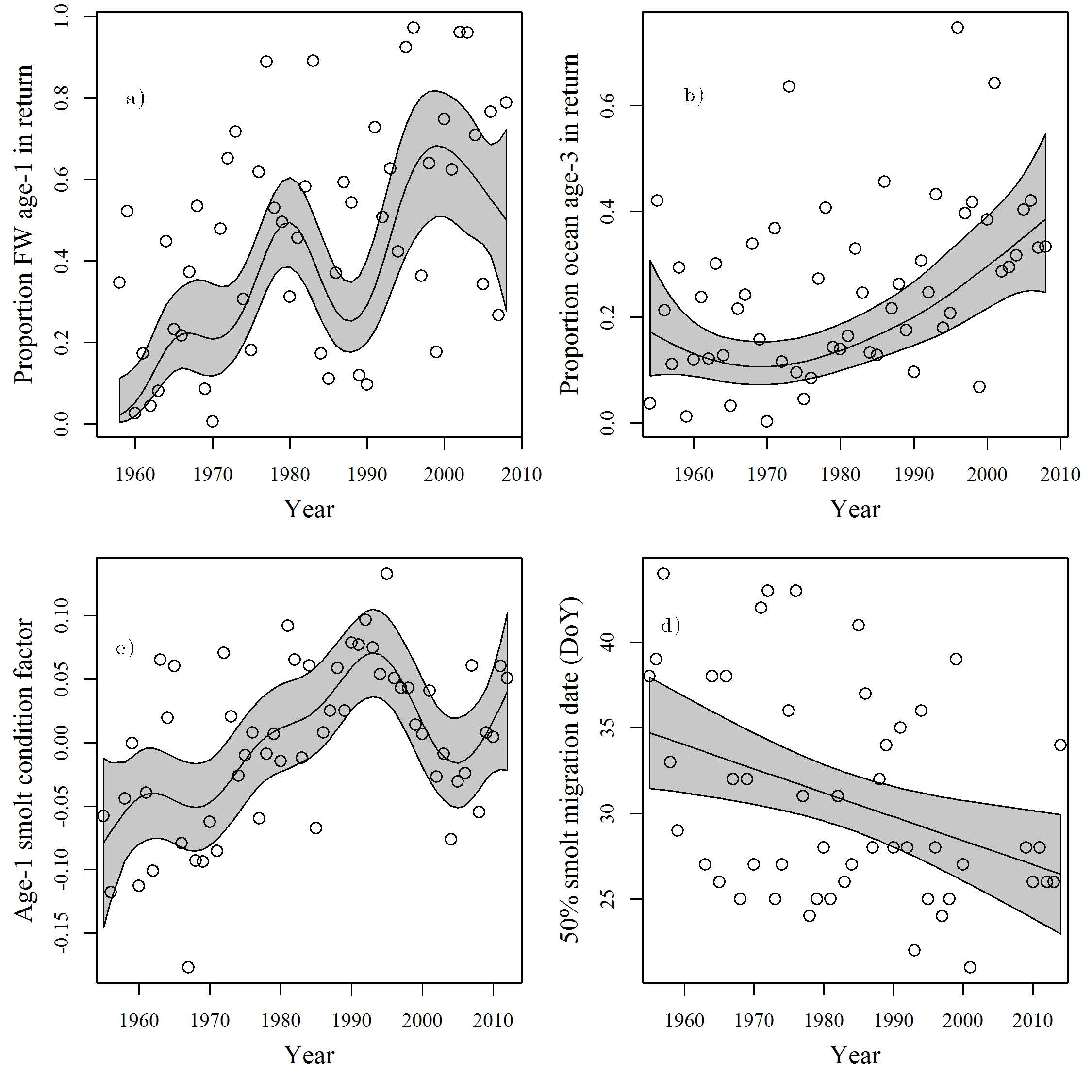

Supplement: S2 Fig — a) Proportion of brood year returning as freshwater age-1, b) proportion of brood year returning as ocean age-3, c) condition factor of age-1 sockeye smolts, d) date on which 50% of total smolt migration is reached. Lines indicate GCV-selected best fit penalized regression spline; shaded areas show 95% confidence intervals and are included to highlight general trends. (TIF) [file pone.0154356.s004.tif]

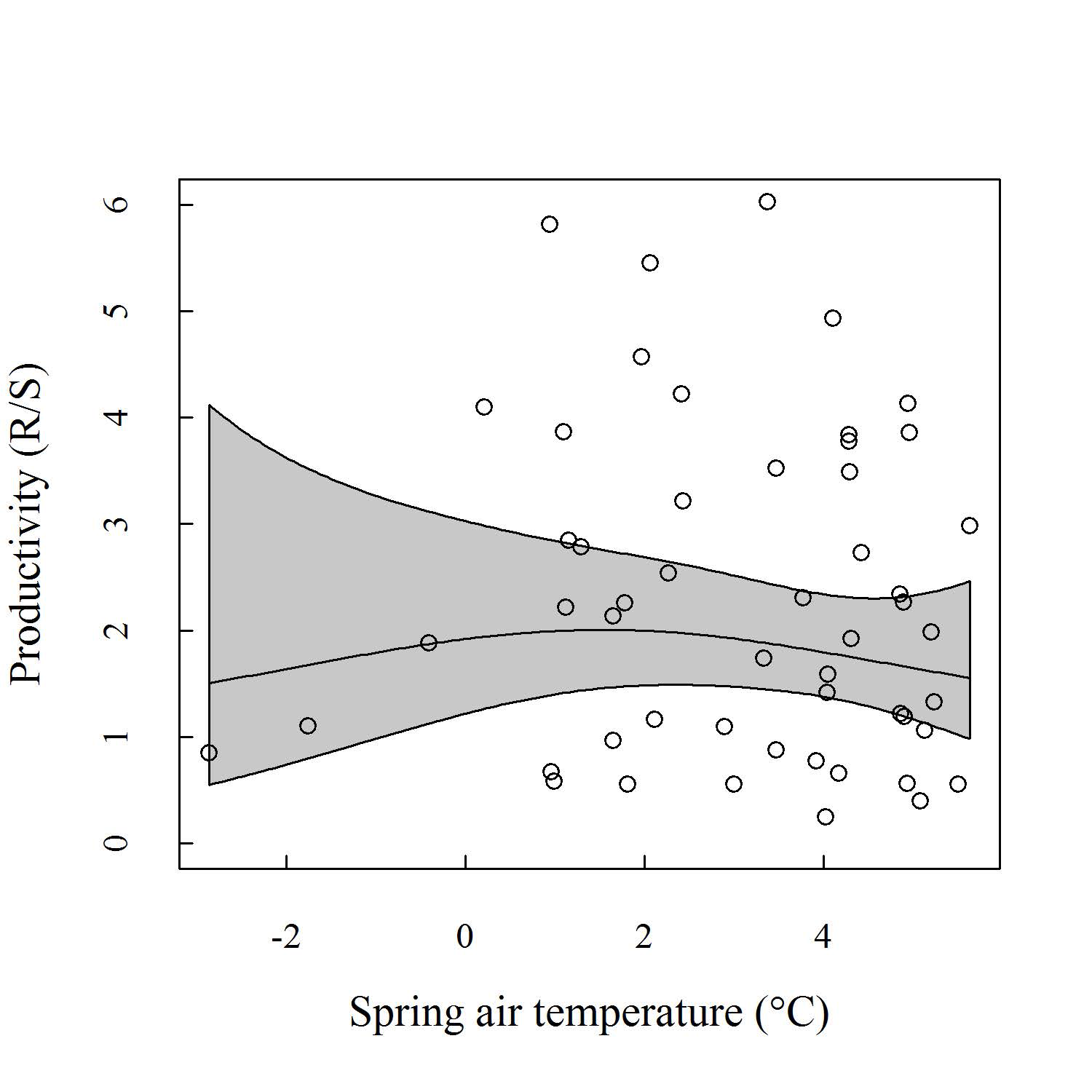

Supplement: S3 Fig — Partial plot of the influence of spring air temperature during freshwater rearing on productivity (R/S). Points show observed data, lines indicates GCV-selected best fit penalized regression spline; shaded area show 95% confidence interval. (TIF) [file pone.0154356.s005.tif]
